# New Trends in Reaction and Resistance to Fire of Fire-retardant Epoxies

**DOI:** 10.3390/ma3084476

**Published:** 2010-08-25

**Authors:** Caroline Gérard, Gaëlle Fontaine, Serge Bourbigot

**Affiliations:** 1 Univ Lille Nord de France, F-5900 Lille, France; E-Mails: cgerard@enscl.fr (C.G.); Gaelle.Fontaine@ensc-lille.fr (G.F.); 2 ENSCL, ISP-UMET, F-59652 Villeneuve d’Ascq, France; 3 USTL, ISP-UMET, F-59655 Villeneuve d’Ascq, France; 4 CNRS, UMR 8207, F-59652 Villeneuve d’Ascq, France

**Keywords:** epoxy, fire-retardant, reaction to fire, resistance to fire, coatings

## Abstract

This paper focuses on current trends in the flame retardancy of epoxy-based thermosets. This review examines the incorporation of additives in these polymers, including synergism effects. Reactive flame-retardants—which are incorporated in the polymer backbone—are reported and the use of fire-retardant epoxy coatings for materials protection is also considered.

## 1. Introduction

Polymeric materials have substituted many materials in our everyday life. Their numerous advantages are also associated to a serious drawback: their poor resistance to fire. Fires cause every year 10 to 20 deaths per million inhabitants in industrialized countries. The number of injured people is ten times higher [1]. Over time, different strategies have been developed in order to enhance the reaction to fire of these materials: use of inherently flame retardant polymers [2], modification of the polymer backbone [3,4] or incorporation of flame retardants into polymers. Because inherently flame retardant polymers can lead to high production costs, the modification of already existing systems is still valued by industries. The modification of the polymer backbone by inclusion of P, Si, B or N often provides good fire properties to the newly synthesized polymer [5]. Even if the range of achievable materials seems unlimited, the obtained copolymer has mechanical properties that are often modified compared to the reference material. Finally, the incorporation of flame retardant particles in polymers is still widely used. It is a simple and cost-effective way for flame-retarding polymers. However, the high levels required for ‘traditional’ flame retardants often induce detrimental modifications of the mechanical properties of the final materials and the use of nanoparticles as flame retardants has been developed. Very different mechanisms are involved in the reaction to fire of polymers containing flame-retardant particles, and these effects are often mixed. Physical actions are encountered when an insulating protective layer is formed on top of the polymer during combustion (intumescent systems) [6], when the degradation of the additive is an endothermic reaction (‘cooling’ effect, e.g., with metallic hydroxides) [7] or when inert gases are released by the additive upon degradation (e.g., calcium carbonate). All these protective ways are associated with a barrier effect. Chemical effects are also involved, for example when the flame retardant or its degradation products disturbs the radical mechanism occurring in the flame and leads to its extinction (halogen-, phosphorus-based flame retardants).

Among the many polymeric materials used, epoxy resins are one of the most problematic: they are used in sectors such as electronics or public transportation, where standards are particularly restrictive. Unfortunately, they tend to burn easily while releasing high quantities of smoke and gases [8]. Because of their use as printed-wire boards, epoxy resins have to be highly flame- and one of the traditional solutions is the incorporation of halogen-containing flame-retardants, especially bromine-based. Part of the standard resin has to be replaced by the bromine-containing monomer, leading to Br contents between 20 to 55 wt %. Furthermore, a higher coefficient of thermal expansion, a higher viscosity and a lower thermal stability are induced by the use of such modified monomers. Another solution is the incorporation of aluminium trihydroxide, but contents up to 65 wt % are necessary in order to get suitable fire properties, resulting in degraded mechanical properties.

Therefore, the effort for enhancing the reaction to fire of epoxy resins has been further developed and innovative solutions have been looked for. This review focuses on the most recent developments in this field and the reported literature has been selected in order to cover the major trends in the flame retardancy of epoxy materials. The first part of this paper is dedicated to the growing use of nanoparticles for enhancing the reaction of epoxies to fire. The current status as well as the difficulties encountered will be reviewed. Then, the evaluation of phosphorus- and silicon-modified resins will be conducted, as well as their possible interactions with nanoparticles. Finally, because of the importance of this application field for epoxies, the developments in flame retardant coatings will be assessed. Considering the modifications induced by the use of carbon or glass fibers (modified heat distribution), reinforced composites will not be considered in this article.

## 2. Incorporation of Additives

**Figure 1:**
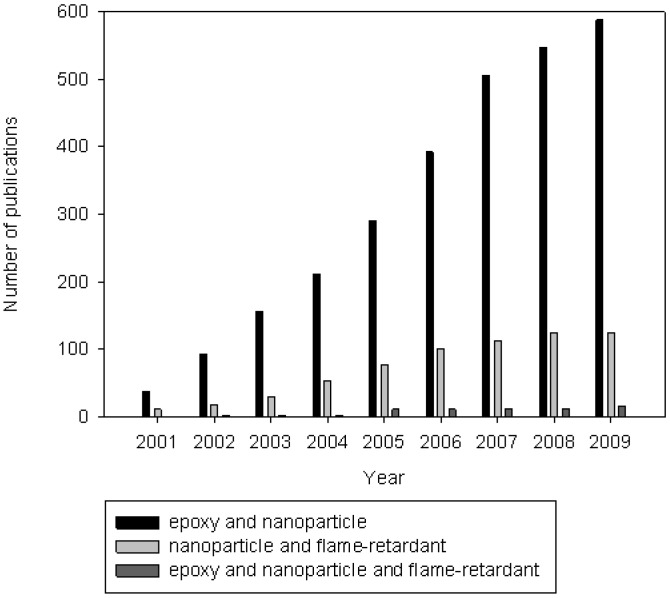
Number of publications per year for different keyword combinations (Scifinder, April 2010).

The additive route has always been widely used to fire-retard polymers, in particular epoxies. It is generally a cheap and easy way of achieving sufficient levels of flame retardancy. However, the traditional additive flame-retarding solutions tend to show their limits. The use of halogen-containing flame-retardants is being questioned: if some of them are suitable for the European market, possible bioaccumulation and effects on workers handling pure substances have been mentioned and environmental issues are still considered. Furthermore, alternatives solutions are required by possible restrictions due to REACH enforcement [9]. REACH stands for Registration, Evaluation and Authorization of Chemicals and this directive from the European Commission will influence the choice of the components used in many areas, in particular in plastics. Metallic hydroxides, such as aluminium or magnesium hydroxide, are efficient at very high loadings (50 wt % or more), but this results in degraded mechanical properties and difficult processing. Therefore, the incorporation of nanoparticles has attracted much interest in the last ten years. Indeed, apart from providing fire properties to the materials, low loadings achieved do not modify the other properties or even enhance some of them, such as mechanical properties [10]. Survey of the literature with SciFinder for different keyword combinations helps to identify the tendencies in the use of nanoparticles. The incorporation of nanoparticles in epoxies for enhancing mechanical properties is widely described (Figure 1, ‘epoxy’ and ‘nanoparticle’). An overview of the literature shows that the occurrences are more limited when coming to the incorporation of nanoparticles in epoxy for flame retardancy (“epoxy” and ‘nanoparticle’ and ‘flame retardant’). However, the use of nanoparticles as flame retardants in other polymers is well-known (‘nanoparticle’ and ‘flame retardant’). The following section is therefore devoted to a screening of the different nanoparticles commonly used as flame retardants, then the results obtained for nanoparticles incorporated in epoxies and the expected developments in this field.

A wide range of nanoparticles has been incorporated in epoxies. The effect of the incorporation of layered silicates, then the potential fire retardant properties of layered double hydroxides, polyhedral oligomeric silsesquioxanes and carbon nanotubes will be discussed.

### 2.1. Organoclays

Among the different nanoparticles used in flame retardancy, layered silicates have attracted much attention in the litterature. As for other nanoparticles, their effect as flame-retardant has been linked to their dispersion state in the matrix [11]. Capability of layered silicates for being exchanged with organic cations at commercial level led to an easier dispersion in various polymers, especially in epoxies. Hartwig *et al.* [12] reported the combustion behaviour of clay-filled epoxies. Two different bentonites based on a phosphonium or an ammonium cation as surfactant were incorporated in an epoxy matrix at 5 wt %. Dispersion studies were conducted by Transmission Electron Microscopy (TEM) and showed a non-uniform distribution of the bentonites in the matrix. For both cations, the morphology is a mixture of intercalated and exfoliated areas, and a slightly better dispersion is obtained with the phosphonium bentonite. The flame-retardant properties of these clays were evaluated by various tests. Limiting Oxygen Index (LOI—ASTM D2863—ISO 4589) gave information about the flammability of the samples: LOI values remain low for nanocomposites and the differences between the virgin polymer and the nanocomposites are not relevant (Table 1).

**Table 1:** Flammability characteristics of clay-filled epoxies. Data from [12].

Samples	LOI (%)	Horizontal burner test Time for burning 150 mm in horizontal position (s)
**Virgin epoxy**	18.3	260
**Epoxy + ammonium clay (4.7 wt %)**	19.7	434
**Epoxy + phosphonium clay (4.7 wt %)**	19.6	464

The flame spread was evaluated by the horizontal burner test (Aerospace standard AITM 2.0003—FAR 25.853), in which the time required for burning 150 mm of the sample is recorded (Table 1). Therefore, the longer the time, the better the behaviour. The time required for burning the sample is drastically increased by the incorporation of bentonites in the matrix. Furthermore, contrary to non-filled epoxy, the nanocomposites do not drip. Finally, the fire behaviour was studied by cone-calorimetry.

After a similar beginning, the curves for the virgin resin and the nanocomposites are different: the first increase is followed by a further increase for the non-filled epoxy, whereas a plateau is reached for the nanocomposites, before a second peak occurs. Different external heat fluxes were used and the influence of the incorporation of nanocomposites depends on the used heat flux. The biggest reductions of the peak of heat release rate (pHRR) are observed for the nanocomposites and are reduced by 1/3 for an external heat flux of 70 kW/m², and no significant difference between the two nanocomposites was observed. The enhancement brought by nanoparticles depends strongly on the heat flux: the reduction of 1/3 at 70 kW/m² is only 1/5 at 30kW/m². Extrapolating this curve leads to an absence of effect for non-radiant tests such as UL-94 or LOI. Therefore, from this point of view, the results obtained before for the LOI test are consistent with the results for the cone-calorimeter. As a first conclusion, the authors pointed out the barrier effect of nanoclays in epoxy. However, in this case, the barrier effect is limited.

Schartel *et al.* [13] also worked on an epoxy system: a bisphenol A diglycidylether-based resin was cured with 4-methylhexahydrophtalic anhydride, and the reaction was accelerated by 1-methyl-imidazole. Different phosphonium-based clays were incorporated at 5 wt % in order to study the influence of the morphology and the distribution of clay in this system: clays with different morphologies were obtained by changing the drying conditions of the exchanged clay obtained by synthesis. The BET surface of modified bentonite was determined by nitrogen adsorption and used to evaluate the powder morphology. It varied from 6.7 m²/g for a commercial ammonium bentonite used for comparison up to 175 m²/g for the freeze-dried phosphonium bentonite. Scanning Electron Microscopy (SEM) and Transmission Electron Microscopy (TEM) showed that the distribution of clay in the matrix is strongly influenced by the characteristics of the clay. It seems that the higher the BET surface of the powder, the better the distribution. Taking into account the results from Hartwig [12], the authors supposed that the incorporation of modified clays alone would not bring sufficient fire properties to the materials. Therefore, they developed mixed systems containing clays and aluminium trihydroxide (ATH). They also used a mixture of unreactive and reactive phosphorus containing flame retardants: triphenylphosphate (unreactive) and Struktol VP3735 (reactive). Struktol VP3735 is the product of the reaction between 9,10-dihydroxy-9-oxa-10-phosphaphenanthrene-10-oxide (DOPO) and an epoxy novolac (no further details available). LOI investigations showed that a small increase is achieved by incorporating clays in the epoxy matrix and the higher LOI is reached with phosphorus-based flame retardants: 23–23.4% depending on the phosphonium clay used, instead of 20.9% for the neat resin and 32.9% for the phosphorus-based flame retardant. It should be noted that combinations between clays and Struktol or ATH led to a small antagonistic effect in terms of LOI. The fire behaviour was further studied by cone-calorimetry: the pHRR was decreased by incorporating layered silicate, ATH or organo-phosphorus flame retardant. The highest decrease is achieved by the organo-phosphorus flame retardant whereas the smallest is reached with clay. The drying conditions of clay, and therefore the morphology, have an influence on the pHRR. As seen before, the dispersion was strongly linked to the drying conditions of the MMT powder and a better nanodispersion leads to reduced pHRR, up to 51% at an external heat flux of 70 kW/m². Another study by Schartel *et al.* [14] on similar systems showed a strong relationship between the clay morphology before mixing and the nanocomposite formation, and thus on the reduction in pHRR. The authors concluded that even if the nanocomposite formation in thermosets in less easy to achieve than in thermoplastics, a homogeneous, nano-scaled dispersion of clay is critical for producing a homogeneous residue surface layer during combustion that is able to provide the desired fire retardancy. Therefore, the need for an even dispersion has been confirmed. However, achieving a perfectly exfoliated nanocomposite is not necessary, and an intercalated structure might provide good fire properties, as long as no big inhomogeneities are present in the material.

More effective systems have been reported by Camino *et al.* [15] with different organo-modified montmorillonite (OMMT) in epoxy at 10 wt %. Such a high loading does not allow the formation of a ‘real’ nanocomposite, *i.e*., a microcomposite is probably synthesized. However, differences in the dispersion state, depending on the exchanged cation, can be observed. The authors showed that the composite containing Nanofil 848 (octadecyl ammonium montmorillonite), which has the best dispersion, observed by TEM and Wide Angle X-Ray Diffraction (WXRD), gives good results but not as good as Cloisite 30B (bis(2-hydroxyethyl) ammonium montmorillonite) (Table 2). On the contrary, Cloisite 25A (dimethyl hydrogenated-tallow(2-ethylhexyl) ammonium montmorillonite) leads to a smaller decrease of pHRR. Such enhancements remain isolated and Zammarano [16] suggested that the particularly high decreases observed for the nanocomposites in this case may be related to different curing conditions between neat resin and the nanocomposites. In fact, 1 wt % imidazole catalyzed the curing for neat resin whereas it was not considered as necessary for the nanocomposites since clays have been reported to catalyze the epoxy ring opening reaction [17]. But it seems that the use of such catalyst decrease the onset of decomposition temperature. If this phenomenon effectively occurs in this case, the materials containing clays cannot be properly compared with the virgin resin. The virgin resin would artificially be easily degraded and the effect of the incorporation of clay overestimated. This could explain why no similar high decreases of pHRR by clays have been reported in epoxy since then.

**Table 2:** Cone-calorimeter data of epoxy-nanocomposites at 50 kW/m² [15].

Sample	Time to ignition (s)	pHRR (kW/m²)	pHRR decrease (%)
**Pure epoxy**	34.5	2,030	-
**Epoxy/Nanofil 848 (10 wt %)**	34.5	1,250	48
**Epoxy/ Cloisite 30B (10 wt %)**	34.5	650	68
**Epoxy/ Cloisite 25A (10 wt %)**	44.0	1,570	23

Even if such high effects are isolated for clays in epoxy, it is worth considering that the higher decrease was not brought by the filler with the best exfoliation. Therefore, the authors mentioned a possible additional effect of the chemical nature of the surfactant in addition to the physical barrier effect of clay. This effect has not been identified up to now. As a general conclusion, it seems that organoclays do not always show their full potential as flame retardants in epoxies, probably because of the difficulties for controlling their morphology in this matrix. However, their barrier effect could be useful in combination with other flame retardants.

### 2.2. Layered Double Hydroxides

Layered double hydroxides (LDHs) are another type of layered crystals and are called anionic clays, by analogy with some lamellar cationic clays. Their incorporation into polymers also provides interesting fire retardant properties: they absorb heat during their decomposition and release water and carbon dioxide, which dilutes flammable gases, enhances the heat absorption and reduces the heat release during combustion. Furthermore, a protective ceramic layer is formed on top of the sample, providing a barrier effect [16]. They could be used as a replacement for magnesium hydroxide and aluminium trihydroxide, which are effective, but need to be incorporated at high loadings. Zammarano *et al*. [18] have noticed the self-extinguishing behaviour of epoxy/LDH nanocomposites at the horizontal UL-94 HB test. High reductions of the peak of heat release rate during cone-calorimeter experiments have also been observed. They depend strongly on the LDH anion: 40% and 51% pHRR decrease are reached with 4-toluenesulfonate and 3-aminobenzosulfonate, respectively. Samples containing montmorillonite were also tested for comparison: a 27% pHRR decrease was observed. Finally, samples based on ‘traditional flame retardants’ such as ammonium polyphosphate (APP) and aluminium trihydroxide were also tested. Even if LDH provided reduced fire behaviour compared to that obtained with APP (80% decrease of the pHRR), the ignition time was close to that of virgin resin for the LDH-containing samples, whereas it was reduced by the incorporation of APP. Furthermore, LDHs are also likely to enhance mechanical properties and APP degrades them. In fact, in this case, it seems that LDHs act as a nano-intumescent system (intumescent systems produce a cohesive and insulating layer on the surface of the polymer, which is blown by the gases produced during the decomposition of the underlying polymer) where the epoxy resin itself is the source of the char, the sulfonate from the LDH is the charring agent, while water and CO_2_ are the blowing agents in addition to those of the degrading epoxy. Therefore, the incorporation of modified-LDH in epoxy seems an effective way for providing good fire properties to this polymer.

### 2.3. Polyhedral Oligomeric Silsesquioxanes

**Figure 2:**
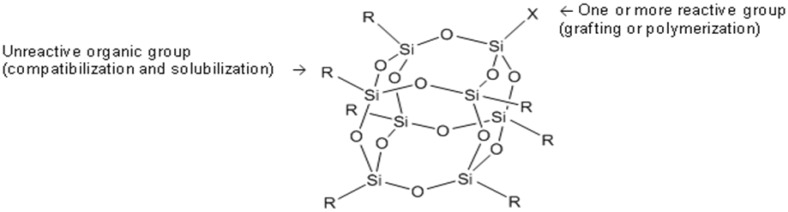
Structure of cage hexahedral silsesquioxane (RSiO_3/2_)_8_.

Among the many particles incorporated in epoxy, Polyhedral Oligomeric Silsesquioxanes (POSS) are one of the most studied in recent years. Their effect on the mechanical and the thermal properties of epoxies as well as their influence on the kinetics of curing has often been reported but the fire properties of the obtained materials have rarely been discussed [19,20,21]. On the other hand, the interest of POSS as fire-retardant in other polymers has been reported [22,23,24]. POSS are structurally well-defined compounds composed of a silicone-oxygen framework having the general formula (RSiO_3/2_)_n_ (*n* = 6, 8, 10…).The most typical species is the octahedron (*n* = 8) (Figure 2).

R can either be hydrogen or any alkyl, alkylene, aryl, arylene or organo-functional derivatives of alkyl, alkylene, aryl or arylene group. These molecules are well-defined and their sizes range from 1 to 3 nm, which makes them the smallest version of colloidal silica. Depending on the reactivity of the organic group, R, POSS can be classified as non-functional or functional.

Functional POSS are widely studied in epoxies, probably because of the possible reactions occurring between their reactive moieties and the epoxy rings of the resin. An enhancement of LOI has been reported by different authors: 5 wt % of cyclohexyltrisilanol POSS leads to a LOI of 32.8% instead of 22.6% for the virgin resin used by Lu *et al*. [25]. Similar trends were observed by Lu *et al*. [26] for cyclohexyldisilanol POSS: the LOI increased from 26% for the virgin resin up to 32% for a POSS content of 25 wt %. Another reactive POSS obtained by the reaction between trisilanolisobutyl POSS and triglycidylisocyanurate was incorporated at higher contents (10 wt %) by Wu *et al.* [27]. Due to the unreacted epoxy ring of the modified POSS, the authors suggested a possible reaction between the hardener and the POSS, leading to a hybrid network. They investigated the reaction to fire using micro-scale calorimetry. The curve corresponding to the hybrid network shows two overlapping peaks instead of one sharp peak and a lower peak of heat release rate compared to the one for virgin resin. Therefore, the peak of heat release rate is decreased by 30%, showing the interest of the incorporation of reactive POSS in epoxies. Even if such loadings appear high for nanoparticles, the incorporation of POSS in the matrix seems beneficial for the flammability. One question still remains: do these results stem from the presence of the reactive moiety of this POSS, the high loading resulting in significant inorganic content or a combination of these parameters?

**Figure 3:**
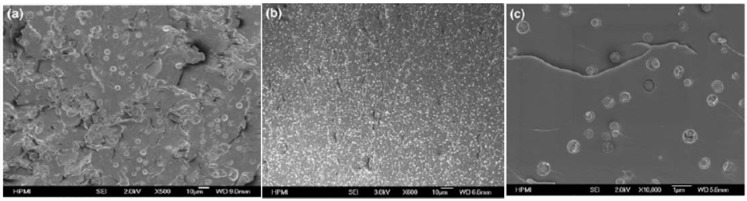
SEM micrographs of POSS composites cured with and without Al. (a) Epoxy/POSS, (b) Epoxy/POSS [Al], (c) higher magnification of (b) [28].

Wu *et al.* [28] studied a DGEBA/phenyltrisilanol POSS mixture. An aluminium complex was used in order to catalyze the reaction between these two reagents. The morphology of the obtained composites was characterized by SEM (Figure 3).

**Figure 4:**
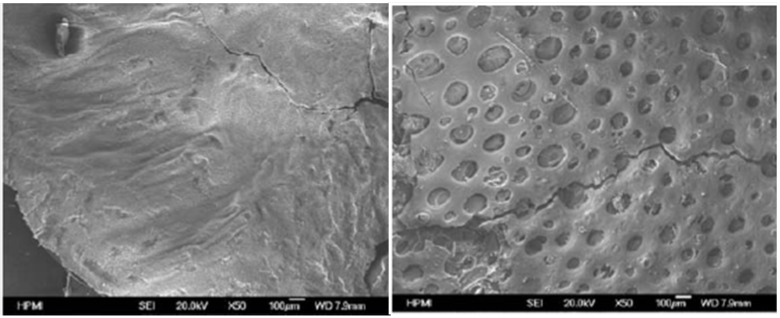
SEM micrographs of residues for Epoxy/POSS[Al] (left) and Epoxy/POSS (right).

The size of POSS particles is decreased when the reaction is catalyzed. In fact, the authors report that the uncured mixture without Al was clear and became cloudy during curing, whereas it remained clear for the samples containing Al. They investigated the effect on the reaction to fire of the resin by cone-calorimetry. All curves are made of two peaks, the first being the highest. The incorporation of POSS in the formulation did not give any significant decrease of the peaks. The effect was higher for the formulation containing both aluminium and POSS: the pHRR was decreased by 33% and the curve is flattened. The morphology of the residues obtained after burning was characterized by SEM: the combination between POSS and Al produces a compact, uniform char, whereas holes were observed when POSS are incorporated without Al (Figure 4).

This difference is probably due to the more even dispersion of POSS when combined with Al. This is important to point out that in this case, the dispersion is not at the nanoscale. However, the addition of aluminium in the formulation led to far more homogeneous dispersion. This is certainly the main reason for the enhanced fire behaviour: increasing the interfacial area between the polymer and the nanofiller helps the formation of a protective layer during combustion. This conclusion is similar to that done by Schartel *et al.* for organoclays [14]. In this case, the authors suggested that the aluminium complex, by favouring the compatibilization reaction between the resin and the reactive moiety of the POSS, leads to the better fire behaviour of the system. However, as a general conclusion on reactive POSS, one does not know if the better reaction to fire observed with reactive POSS is due to the presence of the reactive part or simply to the presence of POSS itself. This last study with the aluminium catalyst showed the potential interest in controlling the reaction between the resin and the POSS in order to take full advantage of the POSS properties.

In order to get higher enhancements of the fire properties, combinations between octavinylPOSS and a phosphorus-containing resin have recently been reported by Wang *et al.* [29]. The phosphorus content in the uncured mixture was kept constant (2 wt %) and the silicon content varied from 0 to 3 wt %. (*i.e*., 0 to 10 wt % POSS). TEM pictures of the sample containing 3 wt % POSS showed a homogeneous material and no aggregate could be identified. The reaction to fire was evaluated by means of a micro-scale calorimeter. The curves are made of a single peak, which is sharp for the resin without POSS. Incorporation of POSS in the matrix leads to a decreased pHRR (−44% for the sample containing 3 wt % silicon) and the shape of the curve is modified, the peak broader. Based on the literature, the authors attributed the positive effect of the incorporation of POSS on the pHRR to the POSS capability for promoting char formation instead of making evolving fuels.

**Figure 5:**
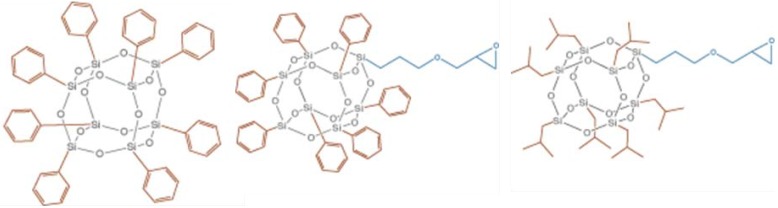
Chemical structures of octaphenyl POSS (left), glycidoxypropyl heptaphenyl POSS (middle) and glycidoxypropylheptaisobutyl POSS (right) [31].

A comparison between reactive and non-reactive POSS was conducted by Franchini *et al.* [30]. Three different POSS were incorporated in an epoxy matrix: octaphenyl-, glycidoxypropyl-heptaphenyl- and glycidoxypropyl-heptaisobutyl POSS (Figure 5).

This selection lets investigate the effect of the non-reactive ligand type and the presence of reactive moieties on the reaction to fire of the samples. Cone-calorimeter at 35 kW/m² was used in this purpose. For each type of POSS, a decrease of the pHRR was observed at 3.7 wt % inorganic part. The phenyl-based POSS led to higher decreases: the reduction of the pHRR was −25%, −34% and −40% for glycidoxypropyl-heptaisobutyl POSS, octaphenyl POSS and glycidoxypropyl-heptaphenyl POSS respectively. Therefore, the phenyl-based POSS led to the highest decreases. The cross-section of the residues was observed and revealed that the sample leading to 40% reduction has a sponge-like structure. This characteristic was not found in the other samples. Such an observation suggests a better thermal insulation and it would be worth observing the residues into details. UL-94 tests were also conducted and the samples were not classified. However, visual observations show differences between the samples: those containing glycidoxypropyl-heptaphenyl POSS did not release drops during burning, 96 wt % of the sample remained after burning and it was self-extinguishing. The other samples burnt completely and released incandescent drops. The effect of POSS content was finally investigated: two different POSS content were used *i.e*., 1.50 or 3.70 wt % of inorganic part. Small differences were observed and the main conclusion is that the performances obtained with 3.7 wt % loading can be reached with 1.5 wt % loading. An influence on the UL-94 test was observed since the final residue was 70 wt % for 1.5 wt % POSS instead of 96 wt % for 3.7 wt %. These results highlight once more the potential of POSS as efficient flame-retardants in epoxy.

### 2.4. Carbon Nanotubes

**Figure 6:**
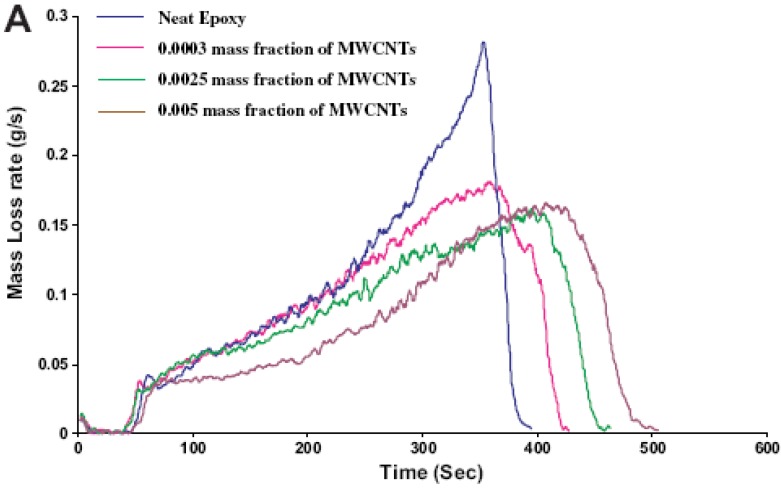
Mass loss rate *versus* time for different CNT-containing epoxies (heat flux: 50 kW/m², N_2_ atmosphere) [35].

Carbon nanotubes (CNT) have been incorporated in various polymers, showing strong fire-retardant effects [24,32,33]. Numerous papers deal with the use of raw or functionalized CNT in epoxy resins and the improvements induced, in particular concerning the mechanical properties and the thermal stability of the prepared materials. However, up to very recently, the effects on the reaction to fire of the resin had never been reported. Kuan *et al.* [34] functionalized CNT with vinyltriethoxysilane and incorporated them in an epoxy matrix via a sol-gel method. Materials containing up to 9 wt % CNT were obtained and tested for their reaction to fire by LOI and vertical UL-94. The LOI gradually increases along with the CNT content and reaches 29% for the samples containing 9 wt % CNT, instead of 22% for the virgin polymer. The UL-94 classification is also increased from V-1 for the virgin resin to V-0 for 3 wt % CNT in the matrix. The authors linked these results to the strongly modified mechanical properties of the materials. The glass transition (Tg) of the polymer is affected by the incorporation of CNT: it increases for CNT contents higher than 3 wt %, but no Tg could be recorded for the sample containing 9 wt %. This result gives a first idea of the potential effect of modified CNT in epoxies. It would however be useful to incorporate unmodified CNT in the sol-gel system in order to observe their effect on the Tg, since it seems closely related to the enhanced behaviour at the UL-94 test. Rahatekar *et al.* [35] studied the effect of highly-aligned CNT in epoxy matrix on the peak mass loss rate (PMLR) during gasification. The system used is similar to a cone-calorimeter, with a nitrogen atmosphere instead of air. Therefore, as pointed out by the authors, the condensed phase is studied in the absence of oxidation and there is no feedback from combustion in the gas phase. The results show the mass loss rate as a function of time. A very small amount *i.e*., 0.0025 wt % of highly-aligned CNT resulted in a 45% reduction of the PMLR and the total time needed for complete mass loss is delayed (Figure 6).

The reduction in PMLR is slightly lower for the 0.005 wt % CNT sample. Based on these results and their modelling, the authors suggest that two mechanisms compete. Re-radiation is identified as the main protection mechanism. The nanoadditives form a protective layer on the surface of the sample. As the temperature rises, the thermal energy reaching the underlying polymer is decreased. At higher loading, this effect is disturbed by the high thermal conductivity brought by CNT: the heat transfer to the degrading polymer below the charring layer increases. Therefore, the fire-retardant effect is less impressive at higher contents. This study provides helpful data for the comprehension of the reaction to fire of epoxies containing nanoparticles. The exceptional results for CNT/epoxy composites might also come from the use of highly-aligned carbon nanotubes, which debundle more easily. This is a major advantage compared to ‘traditional’ carbon nanotubes.

As a conclusion, different nanoparticles have been evaluated in epoxy resins and provide protection against fire. Nanoparticles have already been identified as valuable for the flame retardancy of thermoplastics. One of the involved mechanisms is an increase of the viscosity of the polymer. This effect may be also useful in epoxies since a viscosity drop around 350 °C in epoxy has been identified [36] and could be limited by the incorporation of nanoparticles. However, up to now, the barrier effect is considered as the major effect for protecting this matrix with nanoparticles: an even dispersion of the particles seems essential in order to form a protective barrier on top of the sample. Another conclusion is that for some clays, conclusions are different between the authors. This can be attributed to a wider range of morphology achieved with montmorillonite. For example, working with montmorillonite leads to very different fire-retardant effects depending on the used MMT and the composite synthesis method. Carbon nanotubes have not proven a strong fire-retardant effect in epoxy regarding standard methods yet. LDH seem promising, but the current limited range of industrially available ion-exchange limits their potential use at larger scale. Therefore, until the morphology of MMT/epoxy is controlled, one of the best nanoparticles for enhancing the fire properties of epoxy seems to be POSS, even if they are not nanodispersed at the chosen loadings.

## 3. Reactive Flame Retardants

### 3.1. Phosphorus-Containing Networks

The use of phosphorus-containing flame-retardant has been significantly developed in various polymers as an alternative to halogen-containing flame-retardants [37,38,39]. This is also the case for epoxies. Moreover, due to the multiple possibilities for creating phosphorus-based monomers or hardeners, incorporation of phosphorus directly in the backbone of the resin has been intensively studied, as pointed out by Levchik *et al.* [8,40,41] and Jain *et al.* [42]. The incorporation of the flame retardant directly in the polymer backbone has different advantages, among them avoiding migration of the flame retardant in the polymer before burning. One of the most studied phosphorus reactant is 9,10-dihydro-9-oxa-10-phosphaphenanthrene (DOPO) [40,43,44,45]. It is a commercially available compound and is recommended as part of the curing system of epoxies. However, its main drawback is its monofunctionnality, and therefore it cannot be used as curing agent on its own. Numerous references deal with the chemical modification of DOPO, leading to phosphorus containing epoxies and curing agents [40,46,47]. Only recent works will be commented on in the following section.

Hergenrother *et al.* [48] synthesized different P-containing hardeners and epoxies. The obtained components were used in different formulations. The flammability properties were evaluated by microscale calorimetry, cone calorimetry, Ohio State University (OSU) test and flame resistance (non standard test). In the flame resistance test, the time required for self-extinguishing after removal of the flame is recorded. The reference formulation exhibited self-sustained burning in air but formulations containing the P-containing hardener extinguished immediately. P-content above 0.9 wt % provided a self-extinguishing behaviour. In the case of P-containing epoxies, all the samples containing more than 1 wt % phosphorus extinguished immediately whereas the other burnt between 1 and 2s. Therefore, a minimum P-content in the epoxy or the hardener is required in order to get interesting self-extinguishing behaviour. The reaction to fire was further studied by means of microscale calorimetry. The curves exhibit two peaks, which are shifted to lower temperature when 3 wt % P is incorporated in the systems compared to reference formulations. Furthermore, the height and area of these peaks are also divided by ≈ 2. The authors suggest that similarly to a catalytic mechanism, P reduces both the temperature and activation energy for pyrolysis. The efficiency of P in reducing heat release capacity and enhancing char formation seems to be linked to the oxidation state of phosphorus: PO_4_ >:PO_3_ ≈ RPO_3_ > R_3_PO. Similar conclusions have been drawn by Braun *et al.* [49] on carbon-fiber reinforced composites. Taking advantage of the presence of PO4 acting through a condensed phase mechanism also limits the amount of phosphorus acting in the gas phase. Therefore, the effects in the gas phase, such as a delayed ignition, are less present. This has to be taken into account depending on the final applicability and therefore the standard to pass. Finally, cone-calorimetry confirmed the presence of intumescence for P-containing samples. For some samples, there was even a complete suppression of heat release rate for 1–2 min after ignition. Therefore, this study showed the interest for phosphorus-based epoxies as low-flammable materials.

Combinations between phosphorus-modified resins and nanoparticles have been studied by different authors. Hussain *et al.* [43] synthesized organo-phosphorus epoxy resins by reaction between DOPO and bi- or tetrafunctional resins (bisphenol A diglycidylether –DGEBA- or *N*,*N*,*N*’,*N*’-tetraglycidyl-4,4’-methylenedianiline -TGDDM-), and clay (octadecylammonium-modified montmorillonite, Nanocor I.30) was also incorporated in the systems. DSC measurements showed a catalytic effect of clay on the curing of both DGEBA and TGDDM. Phosphorus-modified resins showed different behaviours depending on the resin type: a decrease in curing temperature, indicating an increased reactivity of the DGEBA system was observed, whereas the contrary was recorded for the TGDDM systems. The authors attributed the difference to the modification of TGDDM with DOPO, which led to a big and stable molecule. The morphology of the composites with clay was also characterized by XRD and TEM. The clay-DGEBA system, either P-modified or not, showed intercalated morphology according to the authors. Clay-TGDDM systems behaved differently: clay in the unmodified resin is exfoliated, and thus better dispersed than in the P-containing resin where an intercalated structure was observed. Therefore, the modification of the resin with phosphorus does not seem to have an influence on the dispersion for the DGEBA systems, but the TGDDM system is affected. The evaluation of the reaction to fire of the systems was evaluated by means of cone-calorimetry at an external heat flux of 50 kW/m² and LOI. Incorporation of 7.5 wt % organoclay or 3 wt % phosphorus in the DGEBA enhances the reaction to fire: the pHRR is respectively decreased by 40% and 50%. However, combination of phosphorus-modified DGEBA and organoclay led to an antagonistic effect since only a 38% decrease was observed. Similar results were found with the TGDDM system: clay alone provides a 17% pHRR decrease, while it is reduced by 52% for 3 wt % phosphorus in the matrix. No synergistic effect was observed for the combination of the two flame-retardants (48% pHRR decrease). LOI experiments led to similar conclusion: the LOI is increased by incorporation of phosphorus or clay in DGEBA, but the combination between the two fire-retardant systems is less efficient. Similar results are obtained for TGDDM. Kiliaris *et al.* [50] recently reviewed these results. They suggested that the lack of synergism between clay and the phosphorus-based resin may be due to the phosphorus moiety hindering the dispersion of clay and leading to a less uniform dispersion. This hypothesis is in accordance with the TEM results and is acceptable.

Liu *et al.* [51] studied further these systems by reacting DGEBA and TGDDM with a phosphorus-containing hardener [bis(4-aminophenoxy)phenyl phosphonate, BAPP] and incorporating the same organoclay at 5 wt % in the resin. Diethyltoluenediamine (DEDTA) hardener was used as reference. An exfoliated or highly intercalated structure for the DGEBA and a more ordered structure, probably intercalated for TGDDM, is claimed by the authors by XRD analysis. However, even if XRD helps to investigate the morphology of nanocomposites, TEM images are required for a definitive conclusion. The reaction to fire was evaluated by cone-calorimetry. Impressive reduction of pHRR is obtained by using BAPP as hardener for both DGEBA and TGDDM systems without clay. In accordance with the conclusions by Hussain *et al.* [43] little evidence of a synergistic effect brought by clay was found. Incorporation of clay in the resins cured with DEDTA showed either as small decrease or an important increase of the pHRR for DGEBA and TGDDM respectively. When the organoclay was combined with the phosphorus-based hardener (BAPP), there was no modification for the TGDDM system whereas a strong antagonistic effect was observed in DGEBA. The incorporation of phosphorus in the epoxy backbone was successfully exploited and the incorporation of nanoclay in epoxy was found to be tricky.

**Figure 7:**
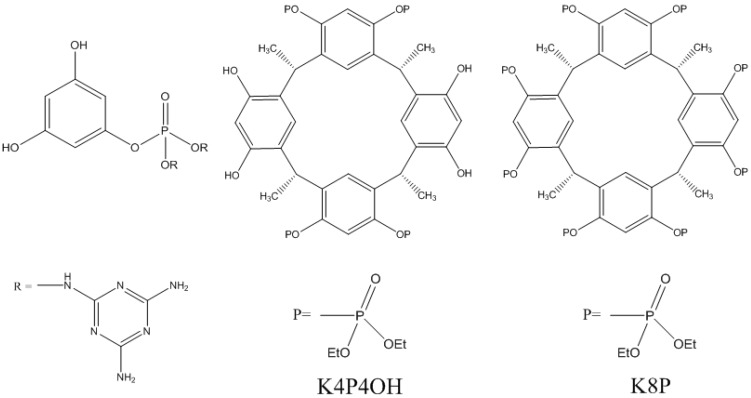
Monophosphorylated phloroglucinol (left), partially (K4P4OH, middle) and fully (K8P, right) phosphorylated C-methylcalix [4] resorcinarene [52].

Toldy *et al.* [52] also studied combinations between phosphorus reactants and clays in epoxy. Two types of molecules, phloroglucinol and calixresorcinarene, were partially or fully phosphorylated (Figure 7).

The best results are obtained with the modified calixresorcinarene. They were identified from the beginning as unable to cure the system and were therefore used as simple additives. Cone-calorimetry revealed that the pHRR is decreased by increasing the quantity of additive. One of the sample corresponding to 2.5% P (named K8P, Figure 7) in the system showed a 61% pHRR decrease. Another additive (named K4P4OH, Figure 7) also provided a delayed pHRR. DSC measurements showed that the reactivity of the system was decreased when K8P or K4P4OH was part of the system. Therefore, the enhancement of LOI is limited (4–5% for K4P4OH and 3–7% for K8P, depending on the filler content). Two types of montmorillonite were then incorporated in the resin at 1–2 wt %: a well-dispersed montmorillonite or the same MMT with K8P intercalated between the clay platelets. However, the enhancements expected from the incorporation of montmorillonite were not reached: the LOI of MMT-K8P is similar that achieved with K8P alone, whatever the clay dispersion. Furthermore the UL-94 classification was degraded from V-0 for K8P alone to HB for the formulations with clay. The authors suggested that this lack of enhancement, compared to thermoplastic matrices, could be due to the crosslinked structure of thermosetting matrices, which hinders the migration of the platelets to the surface and the rapid formation of the protective layer.

Combinations between clays and phosphorus-containing species were further reported by the same group [53]. A phosphorus-containing reactive amine (called TEDAP, without further explanation) was synthesized and combined with montmorillonite and sepiolite for the production of epoxy samples containing 1 wt % clay. The effect of the incorporation of TEDAP in the system was investigated by DSC. The results showed that TEDAP can fully replace the traditional curing agent in this system. Therefore, formulations where TEDAP partially substituted the traditional curing agent were developed and tested for fire resistance. LOI, UL-94, cone calorimeter and glow-wire flammability index showed the superiority of this new curing agent: substituting the original curing agent by 60 wt % TEDAP permits to reach the UL-94 V-0 classification. Total replacement of the original curing agent also led to a LOI value of 33% instead of 21% for the reference resin and the UL-94 classification remains V-0. Furthermore, this formulation showed a 80% decrease of the pHRR and a significant shift in the time to ignition and time to pHRR. It also passed the GWFI test at 960 °C, indicating that this epoxy cured with TEDAP is suitable for electrical equipment. Montmorillonite and sepiolite were then incorporated in the original and the TEDAP-based systems. Slight decreases of the enthalpy of crosslinking were observed by DSC and attributed to the adsorption of the crosslinking agents on the surface of the clay. In the absence of TEDAP, montmorillonite had no effect on the fire properties of the system, whereas sepiolite leads to a small increase of the LOI (from 21% for virgin epoxy to 25%). The combinations between clays and TEDAP seemed moderately interesting since the LOI is increased from 33% for TEDAP alone to 34% for the sepiolite and 36% for the montmorillonite.

Phosphorus-containing epoxies have therefore widely proven their benefit for flame retarding epoxies. The versatility achieved with such molecules opens a wide range of achievable formulations, depending on the final properties needed. Furthermore, sufficient fire properties are achieved with very low P content. Synergies between organoclays and phosphorus-based epoxies have been examined by different authors. However, in accordance to what has been observed in the first part of this paper, the incorporation of clays sometimes helps to enhance fire properties but can also degrade them.

### 3.2. Silicon-Containing Networks

Silicon-based products have proved to be flame-retardant, especially in epoxies. Considering how well phosphorus incorporated in the epoxy networks works, synthesis of hybrids based on epoxy and silicon has been developed. Recent work in this field will be reviewed in the next part: organic-inorganic networks obtained by mixing an epoxy prepolymer with a silicon-containing monomer are often encountered. In order to enhance the reaction to fire, simultaneous incorporation of phosphorus and silicon in the material has also been evaluated.

**Figure 8:**
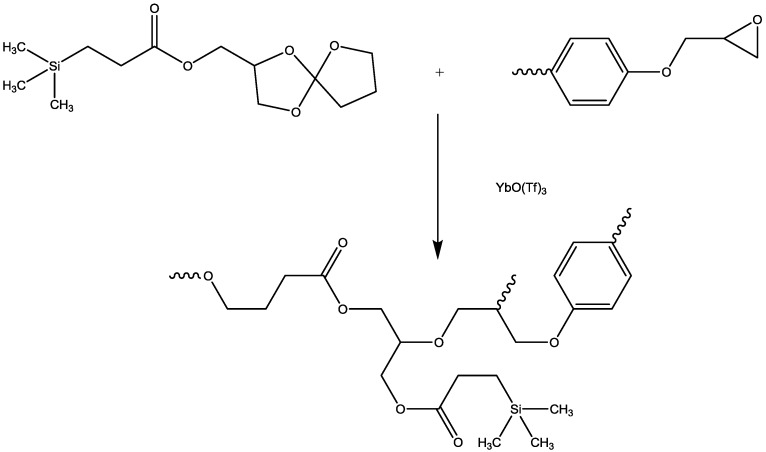
Spiroorthoester containing silicon[56].

Mercado *et al.* [54] reacted DGEBA with diglycidyloxymethylphenylsilane (DGMPS) and cured the obtained resin with diaminodiphenylmethane. Several DGMPS/DGEBA molar ratios were used in order to obtain silicon contents varying from 0 to 8 wt %. It is important to note that the 8 wt % silicon content corresponds to a material based only on DGMPS (without DGEBA). The curing cycle was modulated taking into account the modified reactivity of the system when DGEBA is substituted by DGMPS. Mixed epoxy systems exhibit a single Tg, suggesting a homogeneous material. The flammability was evaluated by means of LOI. It is increased when silicon is incorporated in the epoxy network: 24.1% for the virgin epoxy, 26.1% for the composite made from the 50 wt % DGEBA/50 wt % DGMPS mixture and 36.0% for the sample made from DGMPS only. Even if outstanding values are claimed, the best results are obtained for the sample containing no DGEBA but only the silicon-bearing part of the formulation. The increase for the sample DGEBA50%/DGMPS50% is far less impressive: 2%. The silicon-based resin therefore appears as an alternative to more common epoxy resins. Canadell *et al.* [55,56] synthesized a silicon-containing spiroorthoester and mixed it with DGEBA (Figure 8). DSC showed that the curing exotherm for both homopolymerization occurs at lower temperature than for the mixed system. The curing cycles for the different materials were chosen by taking this into account. Similarly to what was obtained by Mercado *et al.* [54] the Tg of the mixed material is between those of pure materials. LOI helped to evaluate the flammability. The authors reported that measuring the LOI of the material made only from the spiroorthoester was impossible, due to its relatively low Tg (73 °C). LOI of virgin DGEBA was 20%, and that for DGEBA/spiroorthoester was 20.9%.

Harada *et al.* [57,58] studied silsesquioxane derivatives bearing epoxy rings. First, polyglycidyloxypropyl silsesquioxane (PGSQ) was mixed with titanate and cured with DDM. Two different shapes of titanate were used: an organo-layered and a spherical titanate. DGEBA was prepared in the same way for comparison. Horizontal UL-94 tests were conducted in order to evaluate the flammability of the materials. DGEBA/organo-layered titanate and PGSQ/spherical titanate (5 wt %) burnt completely without self-extinguishing, but the PGSQ/organo-layered titanate system extinguished after 8 s and was classified V-0. SEM showed aggregates in the composite containing layered titanate. On the contrary, the spherical titanate showed a uniform dispersion. These results highlight the importance of the shape of the used particles for flame-retardant purpose: different shapes for similar materials can lead to completely different dispersions. In a further study, Harada *et al.* [58] chose poly(glycidyloxypropyl)phenylsilsesquioxane (PGPSQ), mainly because of the reported capability for enhancing flammability properties of phenyl groups. PGPSQ was then mixed with the same organo-titanate as before and cured with DDM. Similarly to what was observed for the PGSQ system, TEM micrographs indicated that a microscopic phase separation occurred. UL-94 horizontal burning test showed that the self-extinguishing behaviour was achieved with only 1 wt % titanate in the PGPSQ system. In order to obtain a hybrid material, DGEBA was mixed with PGPSQ and the titanate. Up to 10–20 wt % DGEBA in the system increases the extinguishing time but the samples are still classified as V-0. For the composite containing 30 wt % DGEBA, no self-extinguishing behaviour could be observed, whatever the titanate content. These studies show the potential of silicon in epoxies. However, the enhancement of the fire performance is often very limited and the best materials are often that containing very small amounts of epoxy resin.

Mixing P- and Si- containing compounds in epoxy matrices have also been considered, but the contribution of Si to the flame retardancy of the systems is not often well identified. Hsiue *et al.* [59] synthesized materials containing P and Si via sol-gel method. A phosphorus-based compound bearing epoxide rings (BGPPO) was mixed with DGEBA and TEOS (tetraethylorthosilicate, often used in sol-gel systems) and cured with DDM. The amounts of BGPPO and TEOS varied. The flammability was evaluated by LOI. First, resins without phosphorus were prepared. The Si content varied in order to identify the effect of Si in the system. The reference resin without Si had a LOI of 26.0%. It increased gradually along with the Si content, up to 31.0% for 8.73 wt % and 10.20 wt % Si. BGPPO alone has a LOI of 36.1% and it is increased for the hybrid material: the LOI reaches 44.5% for the sample containing 4.35% P and 15.63% Si. Therefore, the use of phosphorus-modified epoxy resins is advantageous, and their flame retardancy may be enhanced by the incorporation of small amounts of silicon.

In summary, similarly to what has been done with the incorporation of phosphorus into epoxy backbone, silicon-containing epoxies led to hybrid materials. Limited enhancements of the reaction to fie are obtained by the incorporation of Si. On the other hand, materials mainly based on silion appear as an alternative to epoxy in terms of fire retardancy. Finally, combinations between phosphorus and silicon are promising, with phosphorus being the main flame-retardant and silicon acting as synergist.

## 4. Coatings

Fire-protective coatings are widely used for building, steel or polymer protection. They are often based on intumescent systems. This concept is based on the formation of a protective layer which insulates the underlying material from heat and fire. Such intumescent systems require three major components: an acid source (generally ammonium polyphosphate), a carbon source (such as char forming polymers or polyols) and a blowing agent (e.g., melamine). Chemical reactions between these ingredients lead to the formation of the protective intumescent char. A general mechanism has been widely accepted: the acid source decomposes and yields a mineral acid, then it takes part in the dehydration of the carbon source and finally the blowing agent produces gaseous products. These gases are trapped in the char resulting from the carbon source dehydration and make it swell. In the next section, intumescent epoxy systems, as well as novel approaches like phosphorus-containing UV-curable coatings will be reviewed. Finally, the effect of the incorporation of nanoparticles in intumescent epoxy coatings has never been examined. However, the concept has been tested in acrylate systems and the main tendencies will be presented.

Jimenez *et al.* [36,60,61] widely reported the use of an intumescent protective coating for steel, the formulation optimization and the characterization of its performance. The system is an epoxy-amine system in which two fire retarding agents have been incorporated, separately or combined: a mineral acid (boric acid) and a commercial ammonium polyphosphate. The tests are aimed at linking industrial tests (OTI 95 634) with small-scale lab tests (“Bunsen burner test”). During the industrial test, the sample is heated vertically at high heating rate by a propane burner and the temperature at the backside is recorded. The burning conditions fit as close as possible the ramp of temperature of a hydrocarbon fire heating curve (about 200 °C/min-UL1709). It was observed that the use of the formulation without fire retardants provides properties close to those of the steel plate only. When APP is added to the formulation, an improvement is observed (time of failure 11.3 min compared to 5 min for uncoated steel). However, the char does not adhere correctly to the steel plate and falls off, causing the failure. Similarly, boric acid yields enhanced fire properties (time of failure 18.2 min), but once more, the char falls off the plate. Finally, a combination between these two fire-retardant additives was incorporated into the system and provides the best properties (time of failure 29.5 min) and the char adheres to the plate. The enhanced behaviour was attributed in particular to the combination of phosphates promoting adhesion to steel and of borates which produce a very hard char, resulting in the formation of borophosphates. Experiments using a rheometer were developed and showed that the degradation of the resin at 350 °C leads to a decreased viscosity. The incorporation of boric acid in the formulation limits this decrease by creation of a hard glass (boron oxide), which traps gases and allows a char with good mechanical resistance.

Wang *et al.* [62] reported another trend in epoxy coatings: the use of UV curable epoxy resins modified with phosphorus. They synthesized such a system, tri(3,4-epoxycyclohexylmethyl)phosphate (TECP) and evaluated its fire behaviour by LOI. Various epoxy/TECP ratios were studied. Epoxy alone has a LOI of 21% and TECP alone reaches 35%. As TECP is mixed with epoxy, the LOI increases and reaches 27% for the sample containing 50 wt % epoxy/50 wt % TECP. It reaches 35% for the material based on TECP alone. Mechanical characterizations showed that homogeneous materials are obtained when epoxy and TECP are mixed. Furthermore, the tensile strength of epoxy was improved when TECP was incorporated in it. Therefore, this study suggests that accessing enhanced fire behaviour of UV-curable coatings is possible and can also enhance other properties, such as mechanical ones. Taking into account ecological issues, the use of solvent-free or waterborne coatings will probably lift off, and the production of intrinsically fire-retardant coatings is therefore very promising.

Few publications have recently reported coatings based on epoxy resins alone, but combinations between polyester or acrylates and epoxy resins are found. The influence of the binder in waterborne coatings was studied by Wang *et al.* [63]. An epoxy emulsion was combined with a self-crosslinked silicone acrylate (SSA) emulsion to get a waterborne intumescent coating. In this system, the well-known APP/pentaerythritol/melamine intumescent system is combined with titanium dioxide, kaolin and expandable graphite. Steel plates were coated by five formulations with epoxy/SSA ratios varying from 39/0 to 27.8/11.2 The samples were tested by recording the temperature on the backside of a heated plate. The shape of temperature profiles is similar for all the coatings. At the beginning of the test, there is almost no difference between them, then, after 20 min, a steady state is reached and its temperature depends on the coating formulation: for epoxy/SSA ratios ranging from 39/0 to 33.4/5.6, the temperature is gradually decreased from 270 °C to 243 °C. When the SSA content increases further, the tendency reverses and the temperature reaches 290 °C. Because the efficiency of an intumescent coating is also linked to the morphology of the char, SEM showed that reasonable amount of SSA in the system led to the formation of dense foam. Too big amount of SSA gives rise to large cells, therefore decreasing the insulation, as was observed during the fire test.

Polyester or acrylate intumescent coatings enhanced by nanoparticles have been reported. Their study gives clues about the interest for the incorporation of such particles in epoxies. Wang *et al.* [64,65,66] reported in a series of three papers the effects of nano-LDH and nano-TiO_2_ on intumescent coatings based on mixed acrylate/silicone resins. The aim of the incorporation of these additives was the enhancement of the physical and chemical properties of the char layer, especially adhesion with the substrate. First, a LDH masterbatch was prepared in order to fight the tendency to aggregation of nanoparticles with the aid of dispersant. The dispersion of nanoparticles in the coating was characterized by TEM and compared with the result obtained by the standard procedure. The masterbatch method using dispersant leads to highly enhanced dispersion and was used in the rest of the study. Four coatings containing levels of nano-LDH from 0 up to 3 wt % were tested for their fire-resistant properties on steel. The temperature on the back-side of the sample was recorded by means of a thermocouple. The sample was heated following ISO-834 curve and the fire-resistant time was defined as the time before reaching 300 °C on the backside of the plate. It can be seen that the incorporation of LDH in the coating up to 1.5 wt % gradually increased the protection. However, when the LDH content reached 3 wt %, the tendency reversed. The fire-resistant time of the reference coating is 62 min. Incorporation of 0.5 wt % LDH leads to a similar result but the time reaches 100 min for the coating containing 1.5 wt % LDH. When the LDH content is 3 wt %, the time falls to 48 min. The observation of the residues showed that the intumescence was well-developed for the sample containing 0, 0.5 or 1.5 wt % LDH (char thickness about 18 mm), but it was nearly absent for the higher LDH content (4.3 mm). Various experiments were conducted in order to elucidate this behaviour. The thermal stability of the coatings was then studied by TGA-DTA. The main conclusion is that the incorporation of LDH in this system may enhance the fire protection. Thermal analyses show that the thermal decomposition of LDHs can catalyze the esterification between ammonium polyphosphate and pentaerythritol due to the enhancement of acid activity. XRD showed the presence of aluminium and magnesium oxides. The char containing these oxides seems more resistant and effective than a simple char. LDHs have to be incorporated at a specific content (1.5%): an increase of the LDH content leads to an increased reticulation density, and thus, to a decreased mass loss, but a too high LDH content hinders intumescence. Indeed, incorporation of too much LDH probably increases the viscosity of the system and the formation of the protective layer is more problematic. Furthermore, Castrovinci *et al.* [67] identified an antagonistic effect between ammonium polyphosphate (APP) and aluminium trihydroxide (ATH). APP and ATH reacted together, decreasing the APP amount available for the formation of the intumescent layer. A similar phenomenon could occur for the acrylate intumescent coating since LDH is an organo-modified magnesium-aluminum mixed hydroxide. A further study was conducted on a similar intumescent system containing LDHs (0.9 wt %) and nano-TiO_2_ particles (0.9 wt %). They found that the incorporation of TiO_2_ mechanically reinforced the char by reacting with ammonium polyphosphate or phosphoric acid and forming TiP_2_O_7_. Finally, coatings containing TiO_2_ and no LDH were reported and showed that 1.6 wt % nano- TiO_2_ in an intumescent system already containing micrometric TiO_2_ improves slightly the fire-resistant time from 81 min to 96 min. In this case, nanoparticles are not used as the main ingredients bringing fire properties, since it is incorporated in an intumescent coating. However, they prove their efficiency for enhancing the existing acrylate system when used at an appropriate content. Therefore, it would be interesting to see if such particles bring also interesting properties to epoxy coatings.

As a conclusion, even if fire-resistant coatings are mainly based on ‘classical’ intumescent systems, research in this field is very active and many parameters in the formulation can increase the fire retardancy. Mixing resins, acrylate, silicon or epoxy, opens a new range of available properties. The incorporation of nanoparticles in acrylate systems have shown their potential interest and their transposition to epoxy may be useful. Therefore, depending on the substrate and the use, tailored epoxy coatings can be developed for fire retardancy.

## 5. Conclusions

Current trends in the flame retardancy of epoxy resins have been evaluated. The use of additives still remains a simple way of improving the reaction to fire of epoxy resins. In particular, incorporation of nanoparticles gives the possibility for enhancing the fire properties while maintaining or even improving the mechanical properties of materials. Different particles are used: while carbon nanotubes have not proven yet a strong effect as flame retardants in epoxies, modified layered double hydroxides or polyhedral oligomeric silsesquioxanes turned out to be efficient at low loadings, if not always nanodispersed. The particular case of organoclays is trickier since many factors seem to influence the morphology of the composite and therefore the potential fire-retardant effect.

The incorporation of flame-retarding species such as phosphorus or silicon into the backbone of epoxy resins is also an attractive approach: it provides good fire properties to the matrix while at the same time simplifying the final formulation. Phosphorus is very effective in this type of system at low contents and incorporating it in the epoxy avoids phenomena such as migration during storage. However, it seems that silicon is less effective at bringing suitable fire properties on its own: a synergism between phosphorus and silicon in these systems has been identified.

Finally, the particular case of epoxy coatings was studied. Current systems are mainly based on ‘classical’ intumescent systems. Considering the various available substrates, the different standards to be reached and the presence of new additives, their versatility is still a strong advantage for the development of tailored protective systems.

These three approaches are still promising for flame-retarding epoxy resins. The solution depends on the final properties needed: for bulk materials, nanoparticles show many advantages, among them the low loadings achieved and the often enhanced mechanical properties. However, they have not proven yet on their own their benefit for fire retardancy. Therefore, if chemical modification of the resins is possible for the chosen application, flame retardancy by incorporation of phosphorus into the epoxy backbone appears as the solution providing the highest fire performance. Another wide application of field of epoxies is flame-retardant coatings. Taking into account the different possibilities offered by intumescent systems, sometimes enhanced by nanoparticles, this field still focuses huge interest.
